# A Transcriptomic Survey of Ion Channel-Based Conotoxins in the Chinese Tubular Cone Snail (*Conus betulinus*)

**DOI:** 10.3390/md15070228

**Published:** 2017-07-18

**Authors:** Yu Huang, Chao Peng, Yunhai Yi, Bingmiao Gao, Qiong Shi

**Affiliations:** 1 BGI Education Center, University of Chinese Academy of Sciences, Shenzhen 518083, China; huangyu@genomics.cn (Y.H.); yiyunhai@genomics.cn (Y.Y.); 2 Shenzhen Key Lab of Marine Genomics, Guangdong Provincial Key Lab of Molecular Breeding in Marine Economic Animals, BGI Academy of Marine Sciences, BGI Marine, BGI, Shenzhen 518083, China; pengchao@genomics.cn; 3 Hainan Provincial Key Laboratory of Research and Development of Tropical Medicinal Plants, Hainan Medical University, Haikou 571199, China; gaobingmiao1982@163.com; 4 Laboratory of Aquatic Genomics, College of Life Sciences and Oceanography, Shenzhen University, Shenzhen 518060, China

**Keywords:** conotoxin, ion channel receptor, transcriptome, Chinese tubular cone snail

## Abstract

Conotoxins in the venom of cone snails (*Conus* spp.) are a mixture of active peptides that work as blockers, agonists, antagonists, or inactivators of various ion channels. Recently we reported a high-throughput method to identify 215 conotoxin transcripts from the Chinese tubular cone snail, *C. betulinus*. Here, based on the previous datasets of four transcriptomes from three venom ducts and one venom bulb, we explored ion channel-based conotoxins and predicted their related ion channel receptors. Homologous analysis was also performed for the most abundant ion channel protein, voltage-gated potassium (Kv; with Kv1.1 as the representative), and the most studied ion channel receptor, nicotinic acetylcholine receptor (nAChR; with α2-nAChR as the representative), in different animals. Our transcriptomic survey demonstrated that ion channel-based conotoxins and related ion channel proteins/receptors transcribe differentially between the venom duct and the venom bulb. In addition, we observed that putative κ-conotoxins were the most common conotoxins with the highest transcription levels in the examined *C. betulinus*. Furthermore, Kv1.1 and α2-nAChR were conserved in their functional domains of deduced protein sequences, suggesting similar effects of conotoxins via the ion channels in various species, including human beings. In a word, our present work suggests a high-throughput way to develop conotoxins as potential drugs for treatment of ion channel-associated human diseases.

## 1. Introduction

Cone snail (*Conus* spp.) is the common name for a large genus of small to large-sized predatory marine gastropod molluscs that feed on a variety of prey, including worms, other molluscs, and even small fish [1,2,3]. Corresponding to these prey preferences, cone snails are classified into three groups, i.e., the vermivorous, molluscivorous, and piscivorous species [1]. Nowadays, after more than 55 million years of evolution, there are about 700 described cone snail species around the world [4,5]. All cone snails constitute a single, but the largest, genus of invertebrate marine animals, which is the only genus of the Conidae family within the Conoidea superfamily which belongs to the taxonomic class Neogastropoda [6,7]. *Conus* species inhabit throughout tropical, subtropical, and temperate oceans in the Indo-West Pacific region, such as South China sea, Philippines and Australia [8]. Despite the relatively young age of the genus *Conus*, the slow-moving cone snails have developed successful strategies to subdue prey and defence against foes [5,9], including a highly complex cocktail of potent venom components with a great specificity towards a wide range of physiological targets [10,11]. The sophisticated venom apparatus consists of venom bulb, venom duct, and the hollow harpoon-like radula [12,13].

The venom components are produced in the venom duct and pushed into a radula through systolic pressure of the venom bulb, and then discharged into prey through the radula [5,14]. The modified radulae can be fired at the targets in a harpoon-like action and, subsequently, the venom will be discharged into the victim’s circulatory system, interacting with a range of molecular targets in the nervous system and rapidly immobilizing prey or predators in a few seconds [5,10,11,14].

The major bioactive components of *Conus* venoms are a vast array of unique neurotoxic peptides, commonly referred to as conotoxins [13]. Each *Conus* species contains an average of over 100 unique conotoxin transcripts and 1000–2000 various conotoxin peptides [9,13,15,16,17,18]; therefore, up to 70,000 transcripts and 1,000,000 natural conotoxin peptides may exist in the world, which constitute a great potential resource for pharmacological research and development.

The small sizes of conotoxins, generally 10–30 amino acids in length [19], are produced as precursors comprising a highly-conserved N-terminal signal region followed by a less conserved propeptide region and a hypervariable C-terminal mature region [20,21]. Historically, the modified mature peptides were divided into disulfide-rich and disulfide-poor conotoxin groups based on the number of disulfide bond [22], although this distinction is now considered redundant [23]. The disulfide-poor conopeptides, having none or only one disulfide bond, are further subdivided into contulakines, conantokines, conorfamids, conolysines, conopressins, contryphans, conophans, conomarphines and conomaps [24,25]. With multiple disulfide bonds, the disulfide-rich conotoxins are classified based on three criteria, including the signal region for defining superfamilies (capitalized letters with Arabic numerals), the cysteine pattern (number and connectivity) for defining cysteine frameworks (Roman numerals), and the receptor targets and types of interaction for defining pharmacology families (using lower-case Greek letters) [25,26]. Conotoxins are currently classified into a total of 27 gene superfamilies [13,23,27,28] and 13 temporary gene superfamilies for those identified in the early divergent species [28,29,30]. Conotoxins with a similar cysteine network usually carry a similar signal sequence. So far, these distinct cysteine frameworks have been described in conotoxins and they are often considered to be associated with particular pharmacological families [21,28,29,31,32].

According to the wide range of distinct ion channels, receptors, and transporters in the nervous systems and the interaction types (agonist, antagonist, or delayed inactivation), 12 pharmacological families of conotoxins (α-, γ-, δ-, ε-, ι-, κ-, μ-, ρ-, σ-, τ-, and χ-families) have been defined. Please see more details about definitions of each family in Table 1 [25,28]. Due to their exquisite specificity for receptor subtypes, conotoxins are valuable tools in neurological studies. Several conotoxins are being developed as drugs or drug leads [33,34,35,36,37,38]. The most well-known calcium channel blocker, synthetic ω-conotoxin MVIIA, was approved by the American FDA in 2004 to treat severe and chronic pain [39]. More and more conotoxins are undergoing to be developed for curing many kinds of human diseases, including chronic pain, epilepsy, drug addiction, Parkinson’s disease, and various neurological diseases [40,41].

In our previous report [13], a total of 215 distinct conotoxin transcripts were identified from the vermivorous *C. betulinus*, using both next-generation sequencing (RNA-seq) and traditional Sanger sequencing technologies. In the current study, based on the reported four transcriptomes from the venom duct and the venom bulb, we are interested in exploring ion channel active conotoxins since they play important roles inhibiting or activating ion channel receptors, we also investigated related ion channel proteins and receptors in the transcription datasets. Homologous analysis was performed for the most abundant ion channel protein, voltage-gated potassium (Kv), and the most studied ion channel receptor, nicotinic acetylcholine receptor (nAChR), in different animal species. In turn, conservation of these examples between snails and humans could suggest potential drug development of related conotoxins.

## 2. Results

### 2.1. Summary of the Previously-Reported Transcriptome Sequencing and Achieved Data

Previously, we identified a total of 215 conotoxin transcripts from the Chinese tubular cone snail using five transcriptomes combined with one expressed sequence tag (EST) sequencing [13]. Here, we chose four transcriptome datasets (using the same Illumina sequencing strategy) to explore ion channel based conotoxins and related ion channel receptors/proteins. The four RNA-seq libraries were constructed from three venom ducts (Big, Middle, Small) and one venom bulb (Bulb), in which 4.67, 8.39, 4.58, and 9.77 Gb of paired-end raw reads were generated for each sample respectively. After filtering out low-quality reads, 4.42, 7.95, 4.37, and 9.77 Gb of corresponding clean data were obtained for the followed *de novo* assembly. All transcriptome assemblies were clustered into a single file for redundancy elimination. The final merged assembly contains a total of 300,069 unigenes with an N50 length of 554 bp and a mean length of 428 bp. Please see more details of the transcriptome assemblies in Table 2.

### 2.2. Annotation of Unigenes

To annotate the assembled unigenes, diverse public protein databases were applied in this study, including Nr, Nt, Swiss-Prot [53], Kyoto Encyclopedia of Genes and Genomes (KEGG) [54], Clusters of Orthologous Groups (COG) [55] and Gene Ontology (GO) [56]. Among the achieved 300,069 unigenes, 51,412 (17.13%), 43,885 (14.62%) and 37,392 (12.46%) have matches in the Nr, Nt, and Swiss-Prot databases, respectively; 31,682 (10.56%), 13,981 (4.66%), and 19,276 (6.42%) have hits in the KEGG, COG, and GO databases, respectively. In total, there are 76,317 (25.43%) unigenes functionally annotated on basis of the searched databases (Table S1).

**Figure 1:**
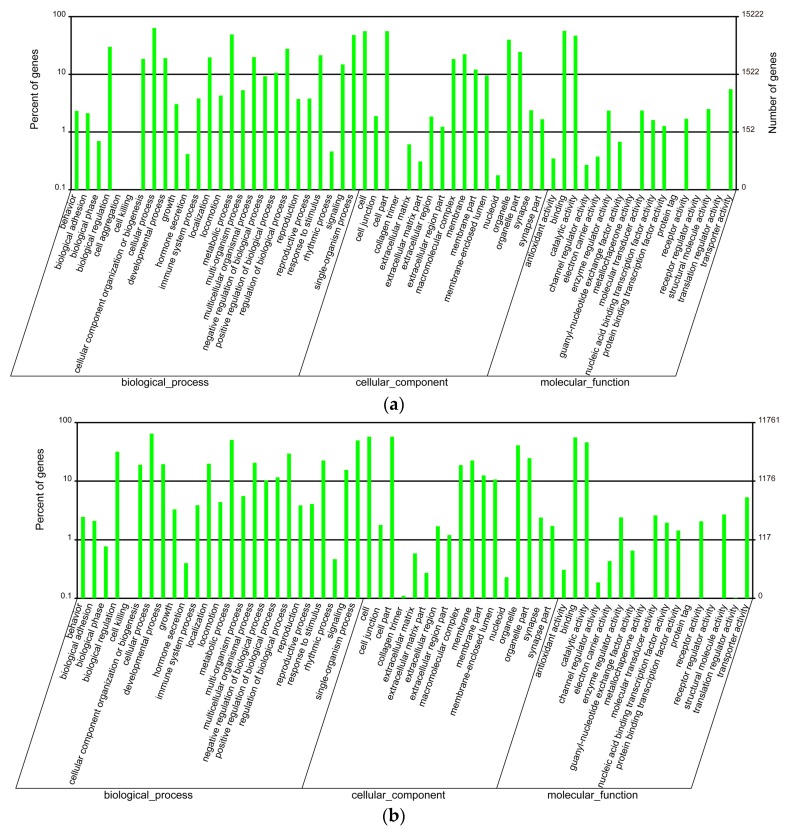
GO enrichment of the annotated unigenes in the venom duct (**a**) and the venom bulb (**b**).

GO enrichment analysis was performed to classify gene functions of these unigenes. Unigenes in each tissue were selected from the merged assembly based on the corresponding clustering results and gene IDs, and then related annotations were applied for GO enrichment analysis. We found that unigenes in the venom duct were enriched into 60 GO classes, in which cellular process, binding, cell and cell part are the top four enriched classes, with 9679, 8621, 8512, and 8511 annotated unigenes, respectively (Figure 1a, Table S2). Interestingly, only one unigene (CL3594.Contig2_All) was classified into the cell aggregation group; while in the venom bulb, annotated unigenes were enriched into 59 GO classes with missing of the cell aggregation term. Likewise, cellular process, cell, cell part, and binding are also the top four enriched terms, although they are slightly different in the order, with 7613, 6764, 6763, and 6579 annotated unigenes in each term respectively (Figure 1b, Table S3). Furthermore, we carried out GO enrichment analysis to classify the achieved conotoxins. In all the reported 215 conotoxins from six datasets [13], 172 conotoxins were identified in the present work due to examination of only four transcriptome datasets (big, middle, small, and bulb). These genes were enriched into 36 GO terms, in which cellular process and metabolic process are the top two with 24 and 22 annotated conotoxins, respectively (Table S4).

### 2.3. Top Highly-Transcribed Channel and Ion Channel Genes in the Venom Duct and the Venom Bulb

Values of reads per kilobase transcriptome per million mapped reads (RPKM) were calculated to illustrate gene transcription levels in each sample (see more details in Table S1). The lack of specific ion channel classification in the COG, GO and KEGG databases urged us to search with the alternative keywords “ion channel” and “channel” against all the annotation results in Figure 1. Both of the keywords have their limitations since “ion channel” narrows the findings by eliminating genes with synonyms or much more sophisticated annotation (such as “ion transport” or “cation-selective channel”), whereas “channel” includes irrelevant genes such as “water channel protein”. Therefore, both keywords were used to obtain a brief and balanced result. Finally, 365 and 1066 hits by searching “ion channel” and “channel” (Tables S5 and S6) were obtained. The searching results were sorted by RPKM values from the highest to the lowest. Top 20 (ion channel) and 50 (channel) highly transcribed genes in each sample (Big, Middle, Small and Bulb) were picked out for comparison.

**Figure 2:**
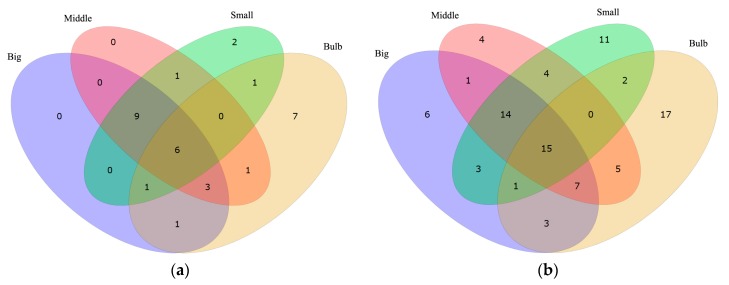
Comparison of highly-transcribed genes tagged with “ion channel” (**a**) and “channel” (**b**) in the venom duct and the venom bulb.

This survey showed that most of the top highly transcribed genes, whether tagged with “ion channel” or “channel”, are the same among Big, Middle and Small samples, while Bulb has other different genes with the highest RPKM values (Figure 2 and Table 3). These data are consistent with the different functions between the venom duct and the venom bulb [13]. In the top 20 “ion channel” genes, the number of individual-specific genes are 0, 0, 2 and 7 in Big, Middle, Small and Bulb datasets, respectively. Similar trend was observed in the top 50 “channel” genes. It seems that the “ion channel” and “channel” proteins transcribed differentially in various tissues. Based on the differential expression strategy of conotoxins in the venom duct and the venom bulb, we agree that cells in the venom duct (the dominant resource of endogenous conotoxins) may have evolved to form special ion channels for various roles of ion channel-based conotoxins.

### 2.4. Identified Conotoxins and Their Predicted Activities

Previously, we observed that most of the 215 identified conotoxins were synthesized in the venom duct while only few were identified in the venom bulb and, interestingly, conotoxins in the bulb were also transcribed at very low levels [13]. Here, we further examined those conotoxins identified in the four transcriptomes and conducted a detailed survey of their predicted activities (on the basis of distinct cysteine frameworks and gene superfamilies [29]). The top 20 highly-transcribed conotoxins in each specimen are summarized in Table 3. Interestingly, we predicted that in all the four transcriptome datasets, more than half (big: 10; middle: 11; small: 12; bulb: 12) of the top 20 conotoxins belong to the I2-superfamily and are labelled with κ activity (inhibiting voltage-gated potassium channels, VGPCs) [57,58]. The second superfamily in the top line is putative μ-conotoxins (big: 9; middle: 11; small: 11; bulb: 10), with the ability to block voltage-gated sodium channels (VGSCs) [59,60,61]. Only one or two ρ-, ε-, and τ-conotoxins were found in the top 20s of the big, middle, and small transcriptomes, while none of these families were identified in the top 20s of the bulb.

### 2.5. Ion Channel-Related Proteins and Homologous Analysis

Conotoxins with their diverse activities are proved to target a wide range of ion channels and receptors [62,63,64,65]. This feature has attracted great attentions from researchers to investigate their potential roles and functions for treatment of human diseases [66,67,68]. Here, we picked out the voltage-gated potassium (Kv) ion channels, which interact with the most abundant group (κ-conotoxins) in the top 20s of Table 3, and the widely studied nicotinic acetylcholine receptors (nAChRs) for further evolutionary analysis.

**Figure 3:**
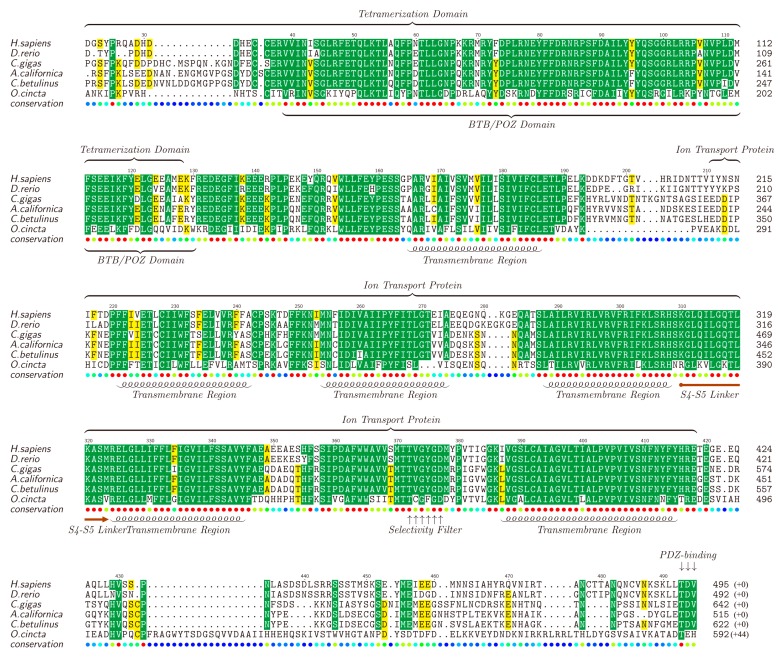
Alignment of Kv1.1 proteins obtained from different species. Yellow blocks show the areas with sequence identity >50% and green blocks indicate the identity >80%.

Kv channels, forming the most diverse group among all types of potassium ion channels, are divided into 12 families (Kv1 ~ Kv12) [69,70]. It is reported that Kv proteins play important regulatory roles and participate in various cellular processes including cell differentiation [71], cell proliferation [72], cell apoptosis [73], functioning of excitable cells [74], and so on. Different types of Kv channel members were identified in the four transcriptomes, including Kv1.1, Kv1.4, Kv2.1, Kv3.1 and Kv5.1, with full or partial sequences. The full-length Kv1.1 was picked out as a representative by chance, translated into the protein sequence, submitted to NCBI (MF179123) and aligned against Kv1.1 proteins of other animals (downloaded from NCBI). The lengths for different Kv1.1 proteins range from 492 to 642 amino acids (see Figure 3).

The results showed that, consisting of about 200 amino acids, the ion transport protein region of the Kv1.1, especially all the functional domains, including five transmembrane helices (TMs), S4S5 linker and selectivity filter, tend to be conserved in all the examined animals. Usually, these Kv proteins contain six TMs [75], and the remaining one TM (not included in the ion transport protein region) is also conserved. Furthermore, the tetramerization domain (T1) is somehow variable in the first 30–40 amino acids while the followed BTB (broad-complex, tramtrack and bric-à-brac) or POZ (*Pox* virus and zinc finger) domain (about 75 amino acids) is highly conserved. The non-conservative segment of the last 50–60 amino acids of Kv1.1 also contains a conserved PDZ-binding site. Existence of the highly-conservative functional domains in these Kv1.1 proteins among different animals suggests similar effects of the putative κ-conotoxins in various species.

**Figure 4:**
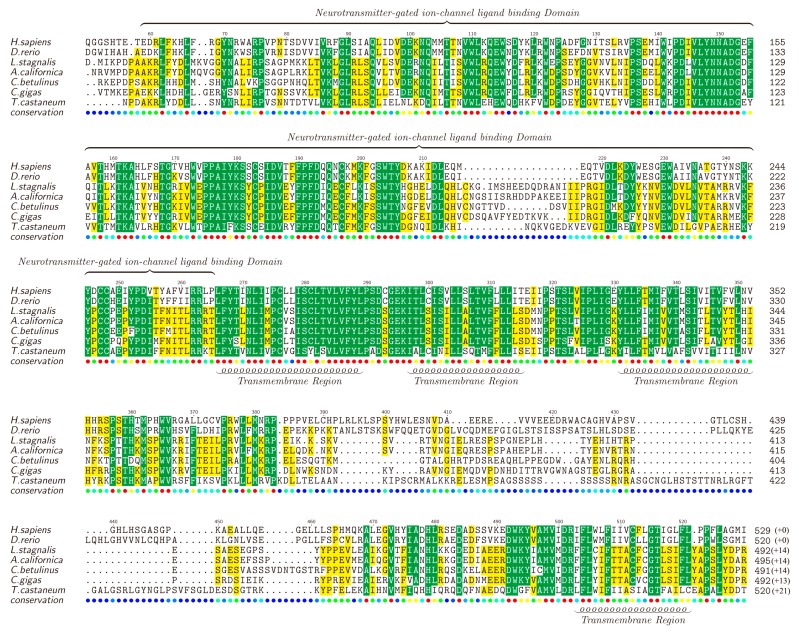
Alignment of α2-nAChRs obtained from different species. Yellow blocks show the areas with sequence identity >50% and green blocks indicate the identity >80%.

nAChRs are a family of receptors involved in modulation of neurotransmission in the central and peripheral nervous system [76,77,78], and have been reported to be associated with diseases such as schizophrenia, Alzheimer’s and Parkinson’s [79,80]. There are many nAChR subtypes, but generally they can be classified into two main groups, the homopentamers (e.g., α7) and the heteropentamers (e.g., α3β4) [81,82]. We also identified various nAChR genes in the examined transcriptome datasets, including α1, α2, α4, α5, α7, α9, α10 and β3. Likewise, the full-length α2-nAChR was selected as a representative by chance, translated into the protein sequence, submitted to NCBI (MF179124) and aligned against α2-nAChRs of other animals (downloaded from the NCBI). The lengths of different α2-nAChRs range from 505 to 541 amino acids (Figure 4).

Similar to the Kv1.1, high similarities were observed in the TMs of α2-nAChRs. It is reported that α2-nAChRs contain four TMs and TM4 is the least conserved [82], which is also verified by us in this study. Furthermore, TM1, TM2, and TM3 are found to be located closely to each other, while there is a long highly-variable loop between TM3 and TM4. In addition, the neurotransmitter-gated ion channel ligand binding domain (LBD), with a length of about 210 amino acids, is conserved in most areas but not in the segment of 210–220 region. Different from the Kv1.1, α2-nAChR seems to be less conserved among the examined animals, but the functional domains still present a high homology, indicating that α-conotoxin activity may partially influence nervous systems of a wide range of diverse species through regulation of the conserved α2-nAChR.

## 3. Discussion

Combination of transcriptome sequencing and the subsequent bioinformatics analysis has become a powerful way to discover and predict novel conotoxins from cone snails [14,83,84,85]. Here, we illustrated the differences of ion channel based conotoxins and related ion channel proteins/receptors in *C. betulinus*, based on our previously reported transcriptomic datasets from both the venom ducts (big, middle, small) and the venom bulb (bulb) [13].

In this study, we regenerated a merged transcriptome assembly containing a total of 300,069 unigenes with an N50 length of 554 bp, and 25.43% of them were functionally annotated according to the public Nr, Nt, Swiss-Prot, KEGG, COG, and GO databases. The GO enrichment analysis of the venom bulb and the venom duct demonstrated similar results of the enriched classes and the distribution number of genes enriched in each term, with cellular process, cell, cell part, and binding to be the top four enriched groups. While all 172 conotoxins, reconfirmed in these transcriptomes, were enriched into 36 GO terms, with cellular process and metabolic process to be the top two enriched ones. These similarities and differences provide insights into these unique conotoxins among all the transcribed genes.

The “ion channel” and “channel” genes transcribed differentially in various samples. In both top 20s and top 50s, the bulb contains the most unique tagged genes, but the ducts share few channel proteins, suggesting differences of ion channels existing in the venom duct (the major tissue to encode conotoxin precursors [85,86]) and the venom bulb (to propel venoms out of the duct [87]). It is estimated that special channels in the venom duct may participate in the protective roles of conotoxins, while the venom bulb functions majorly as the aurilave to discharge conotoxins into the prey [5,14]. Therefore, the gene expression differences are consistent with the function differences.

Among the top 20 highly-transcribed conotoxins in the venom duct and the venom bulb, κ-conotoxins are predicted to be the most common in all samples, followed by putative μ-conotoxins. It seems that these putative κ-conotoxins may have the highest total concentration in the venom mixture of the studied cone snail. Therefore, we extracted the Kv1.1 out from the transcriptome datasets, as well as the most examined α2-nAChR, to act as representatives for further homologous analysis. We observed that all TMs in both Kv1.1 and α2-nAChR are conserved. T1 domain in Kv1.1 is somehow variable in the first 30–40 amino acids, but highly-conserved in the followed BTB/POZ domain, which mediates homomeric/heteromeric dimerization [88]. α2-nAChR contains a conserved neurotransmitter-gated ion channel LBD, which plays important role in the binding of ligands [89], but the TM3–TM4 loop is highly variable. Some studies have shown that this loop forms the largest part of the intracellular domain (ICD) and has special activities [90,91]. The conservation of the neurotransmitter-gated ion channel LBD domain in all species makes the binding of α-conotoxin to α2-nAChRs (from different species) possible, while the variable ICD makes various reactions in cells possible. These interesting data suggest possible ion channel-based toxic pathways for the majority of conotoxins (from cone snails) to be dangerous to human beings (for conservation of ion channels), which in turn proposes a potential hope for development of novel conotoxin-based marine drugs for treatment of ion channel associated human diseases.

## 4. Materials and Methods

### 4.1. Assembly, Annotation, and RPKM Calculation

The generated transcriptome raw data from our previous report [13] (NCBI SRS1009725, SRS1009726, SRS1009728, and SRS1009729) were filtered with SOAPnuke [92] to obtain high-quality clean reads. Reads with adaptors, more than 10% of non-sequenced bases and more than 50% of low-quality bases (base quality score ≤ 10) were discarded. The remaining clean data were then separately *de novo* assembled into contigs and unigenes with an optimized k-mer length of 25 using Trinity software (v2.1.1) [93]. TGICL (v2.0) [94] was used to eliminate the redundancy in each assembly. The final assembly was submitted for functional annotation based on diverse protein databases including Nr, Nt, Swiss-Prot, KEGG, and COG using BLASTX with the threshold of E-value ≤ 10^−5^. Blast2GO software (v2.8) [95] was used to obtain GO annotation with the Nr BLAST results, and WEGO software [96] was used to achieve the GO classification thereafter. RPKM values of the unigenes were calculated using the Cuffdiff package (FDR < 0.05) of Cufflink software (v2.1.1) [97].

### 4.2. Prediction and Identification of Ion Channel-Related Proteins/Receptors

We applied two methods to predict and identify interested ion channel related proteins or receptors from the four transcriptome datasets. The first method is to dig the potential proteins out according to the annotation results. Functional annotation results of diverse databases were merged together according to the gene IDs. Candidate ion channel-related genes were picked out from the merged data with the commonly used names (both abbreviation and full names). Subsequently, the sequences of those selected genes were submitted to NCBI using the tBLASTp tool for further verification. The correctly annotated genes were translated into peptide sequences.

Another method is based on homology search with those reported ion channel related proteins or receptors. We downloaded the interested sequences from the NCBI Protein database. Using these sequences as the queries and our assembled transcriptomes as the local database, we applied BLASTX with the threshold of E-value ≤ 10^−5^ to run the queries against the database. Likewise, the best hits of unigenes were submitted to NCBI using tBLASTp tool for further verification and subsequently translated into peptide sequences.

### 4.3. Alignment and Homology of Ion Channel Related Proteins/Receptors

The obtained full-length ion channel related proteins/receptors, whether predicted by us or downloaded from the NCBI (Table 4), were aligned and sorted by MAFFT software (v7.037) [98] based on their identity. Protein alignments were shaded in different colours by the TEXshade package [99]. Spatial structures and functional domains of each protein were presented according to the identification of that protein in NCBI Protein database combined with the PDB [100].

## 5. Conclusions

This is the first report of ion channel-based conotoxins and related ion channel proteins/receptors in the venomous *C. betulinus*. Similar GO enrichment results were proved in the venom duct and the venom bulb, but different channel and ion channel proteins were identified between the two different tissues. The most common conotoxins with highest transcription levels in the transcriptome datasets are predicted to be κ-conotoxins, followed by putative μ-conotoxins. The ion channel-related protein/receptor, Kv1.1 and α2-nAChR, are conserved in their functional domains, suggesting similar ion channel-based effects of conotoxins in various animal species including human beings, thus making conotoxins a valuable resource for the discovery and development of novel marine drugs to treat ion channel associated human diseases.

## Figures and Tables

**Table 1:** Pharmacological families and related definitions of classified conotoxins [28].

Family	Definition	Reference
α (alpha)	Nicotinic acetylcholine receptors (nAChR)	[42]
γ (gamma)	Neuronal pacemaker cation currents (inward cation current)	[43]
δ (delta)	Voltage-gated Na channels (agonist, delay inactivation)	[44]
ε (epsilon)	Presynaptic Ca channels or G protein-coupled presynaptic receptors	[45]
ι (iota)	Voltage-gated Na channels (agonist, no delayed inactivation)	[46]
κ (kappa)	Voltage-gated K channels (blocker)	[47]
μ (mu)	Voltage-gated Na channels (antagonist, blocker)	[48]
ρ (rho)	Alpha1-adrenoceptors (GPCR)	[49]
σ (sigma)	Serotonin-gated ion channels (5-HT3R)	[50]
τ (tau)	Somatostatin receptor	[51]
χ (chi)	Neuronal noradrenaline transporter	[49]
ω (omega)	Voltage-gated Ca channels (blocker)	[52]

**Table 2:** Summary of the transcriptome assemblies.

	Sample	Venom Duct			Bulb	All
Unigene		Big	Middle	Small		
Total Number		94,026	52,387	114,057	124,004	300,069
Total Length		37,880,261	23,128,493	44,918,779	67,451,577	128,471,163
Mean Length		403	441	394	544	428
N50		413	464	398	681	554

**Table 3:** Top 20 highly transcribed conotoxins in the big, middle, small, and bulb datasets.

Big			Middle			Small			Bulb		
Conotoxin	RPKM	Predicted Activity ^1^	Conotoxin	RPKM	Predicted Activity	Conotoxin	RPKM	Predicted Activity	Conotoxin	RPKM	Predicted Activity
Bt035	84,466.31	NMDARi ^2^	Bt055	77,776.43	κ	Bt035	58,816.45	NMDARi	Bt070	179.21	α, ι, κ, μ
Bt057	72,563.89	κ	Bt018	71,975.85	unknown	Bt018	32,503.63	unknown	Bt035	143.27	NMDARi
Bt018	57,233.21	unknown	Bt082	57,532.96	unknown	Bt075	25,533.62	α, ι, κ, μ	Bt055	106.38	κ
Bt005	21,270.26	α, ρ	Bt035	53,262.22	NMDARi	Bt055	20,861.06	κ	Bt141	91.35	δ, γ, κ, μ, ω
Bt082	20,710.97	unknown	Bt213	43,598.05	ε, μ, τ	Bt005	19,553.90	α, ρ	Bt145	77.8	δ, γ, κ, μ, ω
Bt055	19,799.15	κ	Bt013	38,601.60	NMDARi	Bt070	11,309.30	α, ι, κ, μ	Bt043	56.26	δ, γ, κ, μ, ω
Bt087	11,394.34	α, ι, κ, μ	Bt076	27,361.60	α, ι, κ, μ	Bt043	11,144.72	δ, γ, κ, μ, ω	Bt017	51.57	NMDARi
Bt043	10,995.23	δ, γ, κ, μ, ω	Bt072	25,964.06	α, ι, κ, μ	Bt213	10,602.62	ε, μ, τ	Bt041	42.9	unknown
Bt200	7552.24	unknown	Bt071	22,016.06	α, ι, κ, μ	Bt111	9823.73	unknown	Bt076	34.75	α, ι, κ, μ
Bt044	6067.80	δ, γ, κ, μ, ω	Bt077	21,344.69	α, ι, κ, μ	Bt057	9268.87	κ	Bt005	34.05	α
Bt186	5760.52	δ, γ, κ, μ, ω	Bt125	18,909.36	unknown	Bt013	8930.31	NMDARi	Bt018	28.91	unknown
Bt213	5745.23	ε, μ, τ	Bt145	18,505.50	δ, γ, κ, μ, ω	Bt210	6581.46	ε, μ, τ	Bt186	27.08	δ, γ, κ, μ, ω
Bt141	4616.86	δ, γ, κ, μ, ω	Bt185	17,584.41	δ, γ, κ, μ, ω	Bt081	5527.25	α, ι, κ, μ	Bt054	16.35	κ
Bt075	4512.67	α, ι, κ, μ	Bt043	14,968.06	δ, γ, κ, μ, ω	Bt082	3706.28	unknown	Bt044	15.82	δ, γ, κ, μ, ω
Bt081	4368.21	α, ι, κ, μ	Bt192	12,120.02	unknown	Bt086	3465.02	α, ι, κ, μ	Bt075	15.36	α, ι, κ, μ
Bt100	4154.73	KCb ^3^	Bt075	11,667.17	α, ι, κ, μ	Bt058	3431.21	κ	Bt077	13.29	α, ι, κ, μ
Bt042	4079.54	unknown	Bt141	11,364.97	δ, γ, κ, μ, ω	Bt087	3429.93	α, ι, κ, μ	Bt150	11.54	δ, γ, κ, μ, ω
Bt138	3860.72	δ, γ, κ, μ, ω	Bt136	10,248.83	δ, γ, κ, μ, ω	Bt186	3353.26	δ, γ, κ, μ, ω	Bt100	10.06	KCb
Bt041	3662.06	unknown	Bt005	9612.27	α,ρ	Bt072	3110.04	α, ι, κ, μ	Bt048	9.73	ι
Bt172	3633.23	γ	Bt040	8273.34	unknown	Bt044	2827.06	δ, γ, κ, μ, ω	Bt020	8.01	NMDARi

^1^ The Greek letters denote pharmacological families as conotoxins sharing the same receptor specificities; See more details about related definitions in the ConoServer website [29]; ^2^ NMDARi: NMDA receptor inhibitor; ^3^ KCb: potassium channels blocker.

**Table 4:** Sequences used for the alignments of ion channel proteins.

Gene Name	Genbank Accession No.	Species
**Kv1.1**	NP_000208.2	human (*Homo sapiens*)
	XP_005163101.1	zebrafish (*Danio rerio*)
	NP_001191634.1	sea hare (*Aplysia californica*)
	MF179123	cone snail (*Conus betulinus*)
	XP_011413619.1	oyster (*Crassostrea gigas*)
	ODM96669.1	springtail insect (*Orchesella cincta*)
**α2-nAchR**	EAW63552.1	human *(Homo sapiens*)
	NP_001035417.1	zebrafish *(Danio rerio)*
	NP_001267757.1	sea hare *(Aplysia californica)*
	MF179124	cone snail (*Conus betulinus*)
	XP_011450331.1	oyster (*Crassostrea gigas*)
	ABA60382.1	great pond snail (*Lymnaea stagnalis*)
	NP_001103423.1	darkling beetle (*Tribolium castaneum*)

## Supplementary Materials

The following documents are available online at www.mdpi.com/1660-3397/15/7/228/s1. Table S1. Annotation of all the unique genes and their RPKM values; Table S2. GO enrichment of genes from the venom duct; Table S3. GO enrichment of genes from the venom bulb; Table S4. GO enrichment of conotoxins identified from the transcriptomes; Table S5. Annotated genes tagged with “ion channel” and their RPKM values; Table S6. Annotated genes tagged with “channel” and their RPKM values.
